# A Healthy Vaginal Microbiota Remains Stable during Oral Probiotic Supplementation: A Randomised Controlled Trial

**DOI:** 10.3390/microorganisms11020499

**Published:** 2023-02-16

**Authors:** Anna Lyra, Reeta Ala-Jaakkola, Nicolas Yeung, Neeta Datta, Kara Evans, Ashley Hibberd, Markus J. Lehtinen, Sofia D. Forssten, Alvin Ibarra, Tommi Pesonen, Jouni Junnila, Arthur C. Ouwehand, Keith Baranowski, Johanna Maukonen, Gordon Crawford, Liisa Lehtoranta

**Affiliations:** 1 IFF Health & Biosciences, 02460 Kantvik, Finland; 2 IFF Health & Biosciences, Madison, WI 53716, USA; 3 Oy 4Pharma Ltd., 20520 Turku, Finland; 4 EstiMates Oy, 20520 Turku, Finland; 5 CPS Research, Glasgow G20 7BE, UK

**Keywords:** vaginal colonisation, microbiota, immune markers, lactobacilli, probiotics, *Lactobacillus acidophilus*, *Lacticaseibacillus rhamnosus*

## Abstract

The primary objective of this randomised, placebo-controlled, triple-blind study was to assess whether orally consumed *Lactobacillus acidophilus* La-14 (La-14) and *Lacticaseibacillus rhamnosus* HN001 (HN001) colonise a healthy human vagina. Furthermore, potential effects on vaginal microbiota and immune markers were explored. Fifty women devoid of vaginal complaints (Nugent score 0–3 and vaginal pH ≤ 4.5) were randomised into a 2-week intervention with either La-14 and HN001 as the verum product or a comparable placebo. Vaginal swab samples were collected at baseline, after one and two weeks of intervention, and after a one-week follow-up, for assessing colonisation of the supplemented lactobacilli, vaginal microbiota, and six specific immune markers. Colonisation of *L. acidophilus* and *L. rhamnosus* was not observed above the assay detection limit (5.29 and 5.11 log 10 genomes/swab for *L. acidophilus* and *L. rhamnosus*, respectively). Vaginal microbiotas remained stable and predominated by lactobacilli throughout the intervention, and vaginal pH remained optimal (at least 90% of participants in both groups had pH 4.0 or 4.5 throughout the study). Immune markers elafin and human β-defensin 3 (HBD-3) were significantly decreased in the verum group (*p* = 0.022 and *p* = 0.028, respectively) but did not correlate with any microbiota changes. Adverse events raised no safety concerns, and no undesired changes in the vaginal microbiota or immune markers were detected.

## 1. Introduction

The adult pre-menopausal vaginal microbiota is typically low in diversity and dominated by one or two lactobacilli species or by a mixture of non-lactobacilli genera that form distinct community state types (CSTs), which can be reliably assigned for sample sets originating from various populations [1,2,3]. The CSTs can be defined as CST I (*Lactobacillus crispatus* dominated)*,* CST II (*Lactobacillus gasseri* dominated)*,* CST III (*Lactobacillus iners* dominated), CST IV (non-lactobacilli dominated, such as *Gardnerella vaginalis* and *Atopobium vaginae*), and CST V (*Lactobacillus jensenii* dominated). In addition, hormonal changes during the female lifespan and the menstrual cycle, lifestyle, hygiene practices, and ethnicity can affect the composition and stability of the vaginal microbiota [4,5,6]. Lactobacilli including *L. crispatus*, *L. gasseri, L. jensenii*, and *L. iners* have been associated with a healthy vaginal microbiota [2,7], with *L. iners* also being linked to transitional and dysbiotic stages [7,8]. Non-lactobacilli genera/species, including *Prevotella* spp., *G. vaginalis*, *A. vaginae,* and *Mycoplasma hominis,* are found in a higher abundance when lactobacilli levels are depleted among asymptomatic women and have been associated with bacterial vaginosis (BV) risk [7,9,10].

The vaginal immune system is part of the broader female reproductive tract mucosal immune system that is regulated differently during each phase of the menstrual cycle by the sex hormones [11]. Furthermore, different CSTs, disease states, and genetic variation influence the immunological responses in the vaginal mucosa–microbiota interface. For example, in a cohort of African women, more diverse microbial communities were correlated with higher inflammatory responses [12]. Imbalances of the vaginal microbiota are also characterised by elevated vaginal inflammatory responses. In response to microbiota and hormonal control, the vaginal mucosa secretes a variety of immunomodulatory molecules, including antimicrobial peptides (AMP), protease inhibitors, and immunoglobulins (Ig) [13,14]. One class of AMPs are human β-defensins (HBDs). HBD-1, HBD-2, and HBD-3 are cationic peptides that are active against common vaginal bacteria and pathogens [15]. Secretory leukocyte protease inhibitor (SLPI) and elafin are protease inhibitors which protect host tissue against overactive immune response and harbor antimicrobial and immunomodulatory activity [16]. In some studies, both SLPI and elafin have been reported to be decreased in BV [16,17]. IgA secreted by the cervix plays a key role in microbiota homeostasis on the vaginal mucosa by maintaining commensal bacteria and on the other hand targeting pathogens and preventing their access to vaginal mucosa [18,19].

Probiotic lactobacilli are an attractive and safe approach to support vaginal health as an adjunct or alternative to conventional antibiotic BV therapies [20,21]. Probiotics are defined as live microorganisms that, when administered in adequate amounts, confer a health benefit on the host [22], with the definition permitting both oral and vaginal applications in relation to vaginal health. As a route, oral consumption can be considered more practical and appealing to the consumer, especially in a long-term prophylactic use. Orally consumed probiotics have shown beneficial effects in studies investigating BV (reduced Nugent score) and BV-associated symptoms in comparison to both placebo and conventional antibiotic treatment [23,24,25] and prolonged remission in recurrent BV as adjunct to conventional antibiotic therapy [26]. Vaginal colonisation by orally consumed probiotics has been reported both with strain-level detection [27,28,29] and as elevation of the supplemented species [23,30,31,32]. Additionally, on a community level, an increase in *Lactobacillus* spp. in a dysbiotic vaginal microbiota has been reported even without detectable colonisation in oral, intestinal, or vaginal microbiota samples [33].

In the vaginal tract, probiotics are considered to provide antimicrobial activity by reducing vaginal pH via lactic acid production, producing bacteriocins, and modulating local and systemic immune responses. Furthermore, vaginal probiotics are expected to adhere to vaginal epithelial cells and thereby exclude potential pathogens. However, information is lacking on the effects of probiotic strains on indigenous vaginal microbiota composition and potential immunomodulatory effects in a healthy vaginal tract. The probiotic strains selected for this investigation were *Lactobacillus acidophilus* La-14 (La-14) and *Lacticaseibacillus rhamnosus* HN001 (HN001). La-14 and HN001 included in the Respecta^®^ complex (Giellepi S.p.A. Health Science, Seregno, MB, Italy, containing 5 × 10^9^ colony forming units (CFU) of La-14 and HN001 and 50 mg of bovine lactoferrin per capsule with dosing varying from 1–2 daily capsules) have shown beneficial effects for vaginal health in randomised placebo-controlled clinical trials. These include elevating vaginal *L. acidophilus* and *L. rhamnosus* levels, reducing Nugent score, which reflects an increase in vaginal lactobacilli, and alleviating BV- and vulvovaginal candidiasis (VVC) associated symptoms [23,30,34]. The lactoferrin contained within the Respecta^®^ complex is a glycoprotein able to both inhibit and enhance the growth of Lactobacillaceae and *Bifidobacterium* strains in vitro [35]. Vaginally applied lactoferrin (100 mg and 200 mg in vaginal pessaries for 10 days) has been reported to have a beneficial effect on the vaginal microbiota in a prospective randomised trial [36], and orally consumed lactoferrin (100 mg) may have potential for normalising the vaginal microbiota in comparison to ferrous sulphate as revealed in a pilot study on the use of lactoferrin for the prevention of preterm delivery [37]. However, the bacterial strain components of the Respecta^®^ complex (La-14 and HN001) have been reported to have an alike or enhanced efficacy compared to the combination of La-14, HN001, and lactoferrin in attenuating *G. vaginalis*-induced BV in mice, in inhibiting *G. vaginalis* growth, and in adherence to human HeLa cells in vitro [38], suggesting that lactoferrin is not essential for efficacy. In the mouse study, oral administration was more effective than vaginal application and both systemic and vaginal immune responses were activated [38]. La-14, HN001, and the combination of La-14 and HN001 showed adherence to cervical cells (HeLa cells) and were able to form biofilms in vitro [39]. Moreover, as a pre-requisite for vaginal colonisation after oral consumption, La-14 and HN001 have been reported to survive both through the human gastrointestinal tract [40,41] and the passage from the anus to the vaginal orifice [23,30]. Both *L. acidophilus* and *L. rhamnosus* have long been shown to be safe and suitable for human consumption and have been present in human food for decades [42,43].

The objectives of this clinical trial were to assess whether a two-week oral administration of La-14 and HN001 in a vegan capsule devoid of lactoferrin results in vaginal colonisation of the supplemented species and to characterise the potential effects on vaginal pH, microbiota composition, and immune markers. The target population included healthy premenopausal females devoid of vaginal symptoms, comparable to the study by De Alberti and colleagues [30], with additional Nugent testing at screening to confirm vaginal health with a non-subjective measure. Furthermore, the participants’ menstrual cycle and ethnicity were considered in the study design to minimise confounding variation in the microbiota analyses.

## 2. Materials and Methods

### 2.1. Study Design

**Figure 1:**
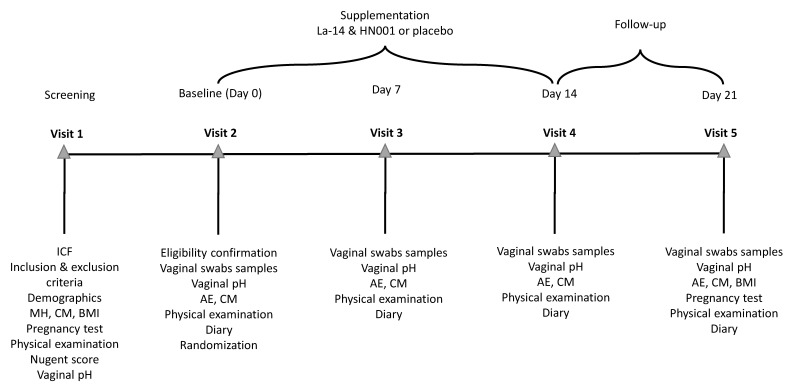
Schedule of events in the study. The screening visit (Visit 1) was held 3 to 42 days before randomisation (Visit 2) to allow for the study endpoints to be measured in between menses. Vaginal pH was measured, and vaginal swabs samples were collected for analysis of vaginal colonisation by the supplemented *Lactobacillus acidophilus* La-14 and *Lacticaseibacillus rhamnosus* HN001 and for assessment of the vaginal microbiota and immune markers at baseline (Visit 2) before investigational product (IP) consumption at weekly intervals during the intervention (Visit 3 and 4) and after a 1-week follow-up (Visit 5). Throughout the study, participants collected information on adverse events, concomitant medications, IP compliance, sexual behaviour, contraceptive method used, and menstrual bleeding days on a diary. A gynecological examination and vital signs’ measurement were performed on each visit as the physical examination. AE, adverse event; BMI, body mass index; CM, concomitant medication; HN001, *Lacticaseibacillus rhamnsosus* HN001; ICF, informed consent form; La-14, *Lactobacillus acidophilus* La-14; MH, medical history.

This was a randomised, double-blind, placebo-controlled, parallel group, single center clinical trial performed at an independent clinical research site, CPS Research (Glasgow, UK), under the supervision of Gordon Crawford, MD, as the principal investigator. The trial included a screening phase, a two-week intervention, and a one-week follow-up with five visits: V1 (Day −42 to −3; screening), V2 (Day 0; baseline and randomisation), V3 (Day 7 ± 1; mid intervention), V4 (Day 14 ± 1; end of intervention), and V5 (Day 21 ± 1; after 1-week follow-up) (Figure 1). The trial was conducted in accordance with the World Medical Association (WMA) Declaration of Helsinki Ethical Principles for Medical Research Involving Human Subjects (64th WMA General Assembly, Fortaleza, Brazil, October 2013) and the International Council for Harmonisation of Technical Requirements for Pharmaceuticals for Human use (ICH) harmonised guidelines for Good Clinical Practice (GCP) E6(R2), dated 9 November 2016, and with laws and regulations for clinical research in the United Kingdom. The trial was registered publicly at the ISRCTN registry prior to initiation (ISRCTN29375062).

After pre-screening, participants attended a screening visit (V1) 3 to 42 days prior to randomisation visit (V2) to allow for visits V2 to V5 to be held in between menses for participants with a natural menstrual cycle. During the screening visit, written informed consent was obtained from all participants before any study-specific procedures or assessments were performed. Screening procedures included assessment of eligibility per set criteria (Table 1), vital signs, a gynecological examination to exclude abnormalities or signs of infection, and collection of demographics (age, ethnicity, smoking, and alcohol consumption), relevant medical history (vaginal symptoms and health, menstrual cycle, pregnancies, relevant operations), height and weight, and conducts potentially affecting the vaginal microbiota (contraception method, sexual activity, multiple or single partners, practice of anal intercourse). To confirm eligibility, a urine pregnancy test, and vaginal swab tests for Nugent score, candidiasis, trichomoniasis, and vaginal pH were performed.

During the study, participants were asked to continue their normal routines and lifestyle (including diet, exercise, choice of contraceptive method), to refrain from sexual intercourse, and use of intravaginal products (lubricants, spermicides) for 24 h preceding visits, and only to use a provided neutral detergent wash for intimate hygiene.

On V2, participants were randomly allocated to receive the investigational product (IP) and instructed to consume one capsule daily after breakfast from Day 1 to the day of V4 (a total of 13 to 15 days). IP compliance was set per protocol at ≥80% and assessed based on the number of returned capsules.

On visits V2 to V5 a gynecological examination was performed, and vital signs were measured. Adverse events (AEs) and updates of contraceptive method, sexual activity, and concomitant medications (CMs) were recorded. For outcome assessments, three vaginal swabs were collected for microbiota and immune marker analyses, and the vaginal pH (NutraBlast^®^ Feminine pH Test Strips, NutraBlast, Pompano Beach, FL, USA) was measured. Between visits, participants collected information on AEs, CMs, IP compliance, sexual behaviour, contraceptive method used, and menstrual bleeding days on a paper diary. In case of menstrual bleeding on Day 20–22, the follow-up visit (V5) was postponed to immediately after the bleeding had ended. Pregnancy test was repeated on V5 as a safety precaution. All AEs were assessed for causality and severity by the investigator.

In case of COVID-19 related restriction to attend a visit on site, the study nurse or investigator contacted the participant, and a remote visit was held including collection of three vaginal self-swabs, AEs, CMs, and IP compliance.

### 2.2. Participants

Premenopausal Caucasian women aged 18–50 years fulfilling eligibility criteria (Table 1) were recruited from the Glasgow (Scotland, UK) area between 10 November 2020 and 24 March 2021.

### 2.3. Intervention

Participants were supplemented either with verum or placebo IP for two weeks. One verum capsule (V-Caps HMPC capsules, Lonza, Puebla, Mexico) contained 10^10^ CFU of La-14 (ATCC SD5212) and HN001 (ATCC SD5675) in a 4:1 ratio, potato maltodextrin as a carrier, and stearate and silicon dioxide as flow agents. The placebo capsules were identical in shape, texture, and taste to the verum capsules and contained potato maltodextrin adjusted to equal the weight of the verum capsule and the same amounts of stearate and silicon dioxide as added to the verum capsule. Both verum and placebo IP were manufactured and packaged using identical containers and labels by Danisco USA Inc. (Madison, WI, USA).

### 2.4. Outcomes

Vaginal colonisation of *L. acidophilus* and/or *L. rhamnosus* at species level was assessed as the primary outcome and vaginal pH was followed as the secondary outcome. Exploratory outcomes included strain-specific vaginal colonisation (La-14 and HN001) and the assessment of vaginal microbiota composition and specific immune markers. AEs were collected as a safety outcome.

### 2.5. Sample Collection and Analysis

Vaginal swabs were collected as dry swabs with the Copan FLOQswab Collection Kit (Copan diagnostics, Murietta, CA, USA) from the sidewalls in the upper third area of the vagina (approximately 5 cm past the introitus) by gently rotating the swab for 10 to 20 s. Before sampling, any excessive secretion or discharge was wiped out and skin contact was avoided. Samples were immediately frozen and stored at −80 °C and all shipments for analysis were conducted on dry ice (temperature range −90 to −20 °C).

#### 2.5.1. DNA Extraction

Vaginal swabs’ flocked heads were clipped directly into a Lysis Matrix E bead beating tube (MP Biomedicals, Santa Ana, CA, USA). The tubes were beaten using a Precellys 24 Homogenizer (Bertin Instruments, Montigny-le-Brétonneux, France), twice for 60 s at 4500 RPM. The bead beating tubes were then spun at 14,000× g for 15 min, then 500 µL of the resulting lysate was carried forward with a FastDNA Spin Kit for Soil (MP Biomedicals) following the manufacturer’s instructions. The purified DNA was quantified using Qubit HS dsDNA kit on Qubit 3.0 Fluorometer (Thermo Fisher Scientific, Waltham, MA, USA).

#### 2.5.2. Detection of Colonisation with Quantitative PCR

The vaginal swab DNA samples were analysed as a primary endpoint with absolute quantitative PCR (qPCR) assays for *L. acidophilus* [44] and *L. rhamnosus* [45], and as an exploratory endpoint for La-14 [46] and HN001. All qPCRs were run with a total reaction volume of 25 µL and 1 ng of sample DNA in triplicate using a QuantStudio 5 Real Time PCR System (Thermo Fisher Scientific), applying either 1 × SYBR FAST or 1 × Taq FAST Advanced master mix depending on the detection chemistry applied (Table 2). Standard curves ranging from 100 fg to 10 ng of La-14 or HN001 DNA were prepared as applicable, and a negative control selected based on optimisation runs (*Faecalibacterium prausnitzii* DSM 17677 for La-14 and *L. acidophilus* DGCC 8698 or *Lacticaseibacillus paracasei* DGCC 4981 for HN001) were included on each 96-well plate. Quantification of PCR amplification was performed using the QuantStudio Design and Analysis Software v1.5.1 (Thermo Fisher Scientific). The limit of detection (LOD) values were based on ½ the number of genomes calculated to be within the 100 fg standard applied. For the SYBR assays, the dissociation curves were analysed to control for non-specific amplification. to control for non-specific amplification.

As a post hoc analysis, an alternative *L. acidophilus* assay previously successfully applied for detection of vaginal colonisation [23,30,47] was tested with a subset of the vaginal swab samples and DNA extracted from pure cultures of *L. crispatus* DSM 20584, *L. jensenii* DGCC 11796, *L. gasseri* ATCC 33323, and *L. acidophilus* strains La-14 ATCC SD5212, DGCC 8698, ATCC 4356, and DGCC 12900.

#### 2.5.3. Vaginal Microbiota Sequencing and Data Analysis

The microbiota populations from vaginal samples were analysed by MiSeq 16S amplicon sequencing of the V4 region (Azenta Genewiz, South Plainfield, NJ, USA) and QIIME2 (v. 2021.2) as previously described [48,49,50] and applied by Lehtoranta and colleagues [7]. Samples containing less than 9000 reads were removed from the study and reverse reads were trimmed at 180 bp and overlapping sequences were paired. Taxonomy was assigned to aligned amplicon sequence variants (ASVs) using ‘q2-feature-classifier’ (classify-sklearn) trained on the V4 region of sequences contained in the RDP Classifier (v. 2.13; training set No. 18 July 2020 release) [51]. Taxa compositions were reported as relative abundance (% of total sequences). Vaginal CSTs were assigned to the samples using the software package VALENCIA [3]. Alpha and beta diversities were analysed as described previously [7] applying Faith’s Phylogenetic Diversity (PD) [52] and weighted UniFrac [53], respectively, and tested with Kruskal–Wallis and permutational multivariate ANOVA (PERMANOVA), respectively, with the latter visualised using principal coordinates analysis (PCoA) with the R (v. 3.4) ‘ggplot2’ package [54,55].

#### 2.5.4. Vaginal Immune Marker Analysis

Immune marker levels (HBD-1, HBD-2, HBD-3, SLPI, elafin and, IgA) were determined by ELISA from the eluants of the vaginal swabs collected from the participants at different time points using the Spectramax 250 ELISA analyzer (Molecular Devices, San Jose, CA, USA). Briefly, the vaginal swabs were reconstituted in ice-cold phosphate buffered saline (PBS) by vortexing and finally the eluant supernatants were aliquoted in Eppendorf tubes for subsequent ELISA analysis. HBD-1 and HBD-3 levels were measured using Human DEFB1 (Beta-defensin 1) and Human DEFB3 (Beta-defensin 3) ELISA Kits (MyBioSource, London, UK), respectively, and HBD-2 by Human DEFb2/DEFB2 ELISA kit (Elabscience). SLPI and elafin levels were analysed with the human ELISA kits (Hycult Biotech, Uden, The Netherlands). IgA was determined using Invitrogen Human IgA ELISA kit. All kits were used according to the manufacturer’s instructions and analysed in duplicate on the Spectramax 250 ELISA analyzer using the software SOFTmax PRO v4.3.1 LS. The lower limit of quantification (LLOQ) was defined as the lowest point on the standard curve for each individual analyte.

### 2.6. Determination of Sample Size

The sample size was calculated using nQuery software (version 8.5.2) with 90% power and a two-sample *t*-test assuming unequal variances and applying a two-sided significance level of 5%. For *L. acidophilus* the mean difference between the verum and placebo arms was assumed to be 60, with standard deviations of 30 (placebo) and 70 (verum) [23]. For *L. rhamnosus*, the mean difference between the verum and placebo arms was assumed to be 30, with standard deviations of 20 (placebo) and 33 (verum) using 10^4^ genome containing particles/100 ng DNA in qPCR results evaluation as the unit for both endpoints for the power calculation [23], although the unit in the analyses was calculated per swab [30] rather than per a mass unit [23] to account for the expected high variance in the amount of host DNA in the samples [56].

With the above assumptions, 40 evaluable randomised participants were needed regarding both primary endpoints. Assuming a 20% attrition rate, 50 participants (25/group) were to be randomised to the study.

### 2.7. Randomisation and Blinding

Study participants were allocated to one of two intervention groups in equal proportions using randomly permuted blocks through a computer-generated process. All verum and placebo products were labelled with a randomisation number accordingly.

All volunteers, study site personnel, clinical CRO personnel, the monitor, and Sponsor laboratory personnel involved in the study conduct were kept blinded during the entire study period.

### 2.8. Statistical Analysis

#### 2.8.1. Primary and Secondary Outcomes

For the primary endpoints, a repeated measures analysis of variance (RM-ANOVA)-model was to be used with fixed effects for supplementation group, repeating factor week (Week 1, Week 2) and the interaction between supplementation group and week. The participant was to be used as a random effect in the models. The contrasts between the verum group and the placebo group at the end of the 2-week intervention period was to be estimated from the model with 95% confidence intervals together with two-sided *p*-values for the null hypotheses. The secondary endpoint (change in vaginal pH) was analysed utilising a mixed effects cumulative logit model, including the same explanatory variables as the models for primary endpoint. A categorised change in vaginal pH (decrease/no changes/increase) was used as the response. The model was constructed to model the probability of lower ordered categories of vaginal pH.

In addition, sensitivity analyses with RM-ANCOVA model and responder analyses, as described in De Alberti et al., [30] were planned for the primary endpoints. No changes in the vaginal pH were expected to occur. No interim analyses of the study data were planned or performed. Intention-to-treat (ITT) population was a priori designated as the primary population of the study. The safety population consisted of all participants who were randomised and received at least one dose of the study product.

#### 2.8.2. Exploratory Outcomes

For the vaginal sequence data, differential taxa were tested for the main effect of group (including model adjustment for visit and random effect of participant or CST at baseline) using the R package ‘ANCOM2’ [57]. Taxa were tested at the family, genus, and ASV level in a participant-paired repeated measures model which included all visits. The analysis considered multiple sampling from each participant. The samples were also subset by visit and modelled separately for each time point. Additionally, differential taxa were evaluated for the main effect of CST at baseline (V2) with model adjustment for visit and random effect of subject. ANCOM2 uses raw ASV sequence counts that have been log-transformed after addition of a pseudocount as input. The log ratios of each taxon were compared to all the remaining taxa one at a time (i.e., ASV1/ASVXX). The test reports a W value analogous to effect size, which represents the number of pairwise tests (taxa ratios) where ASV1/ASVXX significantly differed. The ANCOM2 significance cut-off was set to *p* < 0.05 or *p* < 0.1 after false discovery rate (FDR) correction by the Benjamini–Hochberg method. Spearman correlation analyses were conducted using the R packages ‘hmisc’ and ‘gplots’ for individual visits. *P*-values were adjusted for multiple comparisons by Benjamini–Hochberg FDR as noted.

Differences in CST distribution between intervention groups at baseline and for change in CST by timepoint (V3, V3 and V4 together, V4 and V5) were checked with Fisher’s exact test. CST distribution and ASV correlations were also assessed for demographic variables including Nugent score, age, body mass index (BMI), contraceptive method (hormonal/non-hormonal), and pregnancies (prior pregnancies/no prior pregnancies) with the Chi-Square test and Spearman correlation, respectively. In addition, the percentages of participants with over 1% increase or decrease from baseline in the prevalence of an ASV were tabulated and tested with Fisher’s exact test as an effort to detect effects related to probiotic consumption.

For each immune marker, the differences between verum and placebo in change from baseline were analysed with a RM-ANCOVA-model. The models included fixed effects for supplementation group, repeating factor visit and the interaction between supplementation group and week. Baseline value was included in the model as a covariate. Participant was used as the random effect in the models. The contrasts between verum and placebo at different visits were estimated from the model including 95% confidence intervals (CIs) together with two-sided *p*s. Change from baseline used in the RM-ANCOVA model was calculated using logarithm transformed values. If model assumptions were not met, square root transformation was used when calculating the change from baseline. Model assumptions were confirmed testing residual normality using Shapiro–Wilk test. If normality assumptions were not met with either of the data transformation, nonparametric Wilcoxon rank sum test was applied by visit to compare group differences.

For statistical analysis of microbiota and immune markers, the groups were analysed together. The R package ‘rmcorr’ was used to correlate immune markers (all timepoints joined) and ASVs present in at least 40% of the participants using repeated measures and *P*s were adjusted for multiple comparisons by Benjamini–Hochberg FDR. Additionally, immune marker concentrations were compared with Wilcoxon test between participants grouped according to CST at baseline (regardless of intervention group).

## 3. Results

### 3.1. Participants

**Figure 2:**
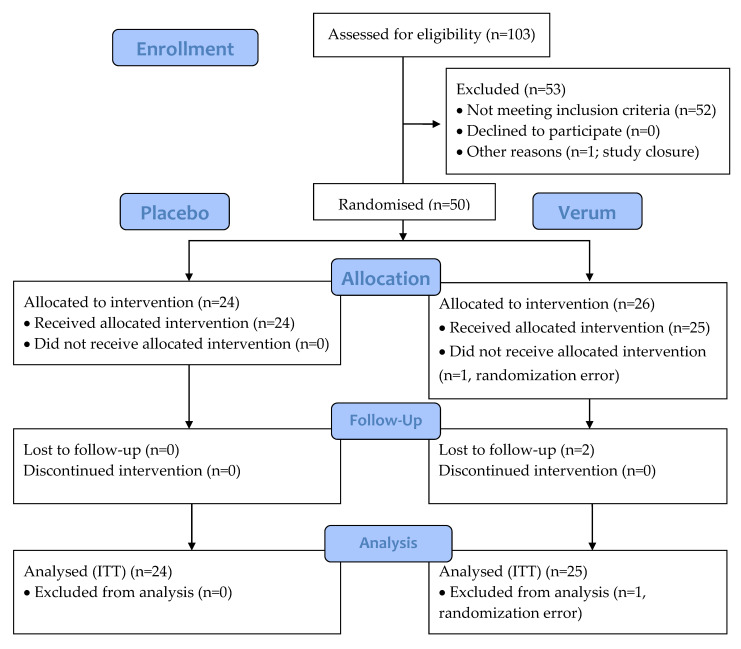
CONSORT flow diagram of the 21-day intervention study of triple-blind, randomised, and placebo-controlled design. Placebo: maltodextrin; Verum: 10^10^ CFU *Lactobacillus acidophilus* La-14 and *Lacticaseibacillus rhamnosus* HN001. CONSORT, consolidated standards of reporting trials; ITT, intention-to-treat.

A total of 50 participants were randomised and 47 participants completed the study. Of the participants, 1 was discontinued due to randomisation error and did not receive any IP, leaving 49 participants in the ITT and safety analyses (Figure 2). The two other discontinued participants were lost to follow-up after the 2-week visit and took at least one dose of study product. The groups were comparable according to demographics, characteristics of menstruation cycle, and obstetric history (Table 3 and Table 4). There were no reports of pain in the lower abdomen, presence of vaginal inflammation, dryness, itchiness, stinging, sterility, or vulvovaginal infections reported in the history of the randomised participants and only one leucorrhea (placebo group) and one pelvic or abdominal pelvic pain (verum group).

### 3.2. Vaginal Colonisation and pH

DNA extraction was successful from all samples, but the efficiency varied considerably, having a range of 206 to 32,400 ng/swab (median 5340 ng/swab, average 8405 ng/swab, SD 7863 ng/swab) for all 188 samples.

For the primary endpoint, there was no detection of *L. acidophilus* above the assay’s LOD 5.29 Log10 genomes per swab in either group at any of the sampling timepoints (Table 5). Two participants in the placebo group showed clear amplification at the follow-up visitV5 for *L. rhamnosus*, although one of them was below LOD (Table 5). The strain-specific qPCR analyses conducted as exploratory outcomes did not detect any amplification above LODs 5.29 or 5.11 log10 genomes per swab for La-14 or HN001, respectively. Thus, no statistical analyses were performed for the primary or exploratory assays on colonisation. IP allocation was confirmed for all participants by culturing samples from the returned IP capsules after the study.

The *L. acidophilus* qPCR assay successfully applied earlier for detecting colonisation [23,30,38] amplified four *L. acidophilus* strains (La-14, DGCC 8698, ATCC 4356, and DGCC 12900) efficiently, but with two peaks repetitively present in the melt curve analysis (a main peak and a shoulder-like second peak). With *L. acidophilus* as standard, 82 °C (dissociation temperature of the main peak) was the dissociation temperature for standard-based quantification of samples in the qPCR analysis. On the other hand, vaginal swab samples (137/141 of the tested samples) and commensal vaginal species *L. crispatus*, *L. gasseri,* and *L. jensenii* all amplified with a uniform amplicon dissociating at 78 °C with a single peak, although PCR-efficiency was poorer. As the amplicon melting point for *L. acidophilus* was 4 °C higher than that of the samples, we were unable to quantify the samples with *L. acidophilus* as standard and had to reject the uniform amplification seen in the samples as background. With *L. crispatus* as standard, quantification of samples was possible and results could be calculated as the number of participants presenting an over 2-fold increase from baseline, as previously conducted by De Alberti and colleagues [30] (Table 6).

Vaginal pH remained stable over the intervention, being 4.0 or 4.5 for at least 95% of the participants during the intervention phase (Supplementary Table S1) and no statistically significant differences in category of pH change (decrease, no change, increase) were found between the intervention groups (Table 7).

### 3.3. Vaginal Microbiota

**Figure 3:**
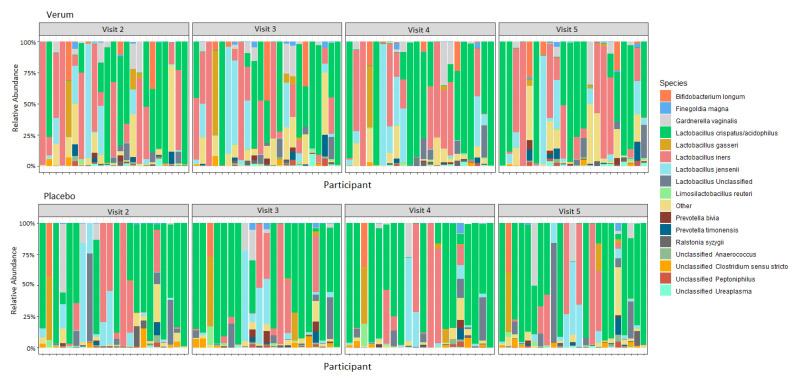
Relative abundance of species in the vaginal microbiota for verum and placebo participants consuming 10^10^ CFU *Lactobacillus acidophilus* La-14 and *Lacticaseibacillus rhamnosus* HN001, or maltodextrin daily for two weeks between Visits 2 and 4. Visit 2, baseline visit; Visit 3, after one week of intervention; Visit 4, after two weeks of intervention; Visit 5, after a one-week follow-up.

A total of 176 samples (90 verum and 86 placebo samples) had at least 9000 reads and were included in the analysis. For both groups and all timepoints, Lactobacillaceae was predominant, with *L. crispatus/acidophilus, L. iners,* and *L. jensenii* being the three most common ASVs within the sequence data (Figure 3; Supplementary Table S2). Community wise, CSTs I (*L. crispatus* dominated), II (*L. gasseri* dominated), and V (*L. jensenii* dominated) were most prevalent (Table 8). There were no changes detected in the relative abundance of microbial families, genera, or ASVs between visits within the verum or placebo groups (ANCOM, *p* > 0.1), nor did any taxa differ when comparing between the groups at any visit including the baseline (ANCOM, *p* > 0.1). Similarly, the baseline CST distribution did not vary significantly between groups (Fisher’s exact test *p* 0.7547).

**Figure 4:**
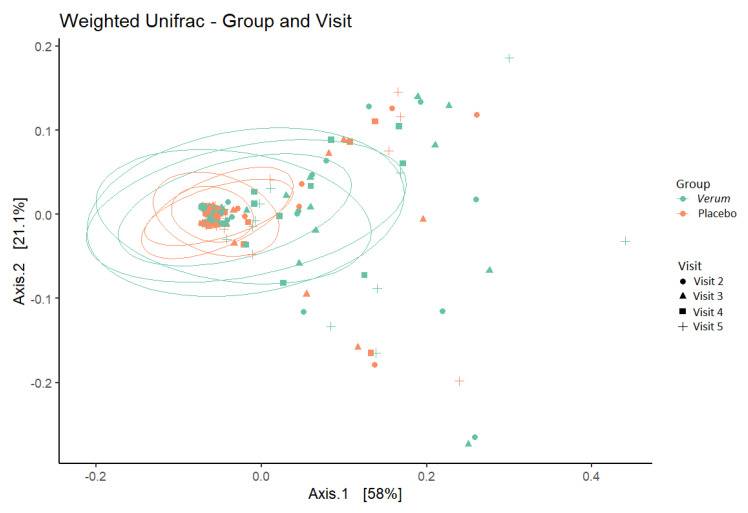
β−diversity (weighted UniFrac metric) depicting sample clustering of the vaginal microbiota samples from study participants by intervention group and visit in a principal coordinate analysis (PCoA) plot. Ellipses represent 95% confidence interval. Visit 2, baseline visit; Visit 3, after one week of intervention; Visit 4, after two weeks of intervention; Visit 5, after a one-week follow-up.

No difference was seen in within-sample species diversity (α-diversity) between groups or timepoints. For between-sample dissimilarity (β-diversity), the percent of variation that could be attributed to the study factors was mainly due to individual (77%) and, to a lesser extent, to CST grouping (10%), intervention (3%), BMI (3%), and Nugent score at screening (1.5%, and age (1.5%), whereas visit (time) did not have a significant influence on the observed diversity (Figure 4).

Abundances of families, genera, and ASVs were comparable between verum and placebo and remained stable over time. One participant from the placebo group, with *L. rhamnosus* also detected in qPCR, had ASVs assigned to *L. rhamnosus*. Table 9 shows the percentage of participants having an over 1% change in the relative abundance of ASVs assigned to *G. vaginalis* and predominant *Lactobacillus* during the intervention. The number of participants with over or under 1% change in ASV abundance did not differ significantly between groups or timepoints (Fisher’s exact test).

With all samples combined (both groups and all visits) the relative abundance of ASVs assigned to *Prevotella, Finegoldia, Campylobacter ureolyticus,* and *Streptococcus anginosus* correlated positively, whereas ASVs within Lactobacillaceae did not show correlation. Of the demographic variables, BMI correlated positively with *Gardnerella vaginalis* and *Campylobacterium ureolyticus* (*p* for Spearman correlation < 0.01 with analyses limited for ASVs present in at least 40% of participants).

### 3.4. Vaginal Immune Markers

For the immune marker analysis (Table 10; Supplementary Figure S1), log-transformed data were used for SLPI, elafin, and IgA, whereas square root transformed data were used for HBD-1 and HBD-3 that did not fulfil the model assumptions for log transformation. HBD-2 had 17%, 39%, 34%, and 31% of the values below the LLOQ for timepoints V2, V3, V4, and V5, respectively, and was thus tested with non-parametric methods using 31.25 pg/mL (LLOQ divided by 2) imputed for the missing values before transformation. There were no significant differences regarding HBD-2 for any of the visits; hence, it is not included in the Table 10.

The RM-ANCOVA analysis showed statistically significant differences between the study groups for elafin (*p* = 0.022) and HBD-3 (*p* = 0.028) over all visits (Table 10). For elafin, the comparisons performed on single visits were not statistically significant (*p*-values ranging from 0.051 to 0.071; however, the *p*-values can be considered as a trend towards significance. The difference appeared to be consistent over all post-baseline visits, in an agreement with the overall statistical difference. In addition, the results indicated a possible similar decreasing trend for another protease inhibitor SLPI (*p* = 0.15 overall and *p* = 0.056 at V5).

HBD-3 also showed an overall statistically significant decrease in verum compared to placebo with an increasing numerical trend in the difference between the groups adjusted for baseline. On the follow-up visit (V5), the difference between the groups was statistically significant (*p* = 0.008).

Correlations were assessed for immune markers and 16 ASVs that were present in at least 40% of the participants (i.e., *Lactobacillus* spp., *L. crispatus*/*acidophilus*, *L. jensenii*, *Limosilactobacillus reuteri*, *L. iners*, *Fenollaria massiliensis*, *Finegoldia magna*, *Streptococcus angiosus*, *Campylobacter ureolyticus*, *Prevotella bivia*, *Prevotella disiens*, *Prevotella timonensis*, *Peptoniphilus* spp., *Rastolnia syzgii*, and *G. vaginalis* presented by two separate ASVs). Statistically significant (*p* < 0.05) correlations were found for HBD-1, HBD-3, and IgA, but not for HBD-2, elafin, and SLPI, as detailed in Table 11; Supplementary Figure S2.

When comparing levels of immune marker for each CST at baseline (verum and placebo grouped combined), a trend of higher SLPI and lower HBD-3 was seen in CST I compared to CST III (Wilcoxon test *p =* 0.0918 and *p =* 0.1221, respectively; Supplementary Figure S3). CST II and IV were not included in the analyses due to the prevalence of them at baseline being too low (Table 8).

### 3.5. Safety

Only four participants had IP compliance < 80% (two in verum and two in placebo). IP stability was confirmed to be above target potency (10^10^ CFU/capsule) before initiating the clinical phase and after the last participant had completed the study. There were no differences in vital signs, menstruation, or vaginal health between groups during the study. After randomisation, only one participant reported reddening of vulva and/or vagina (verum group). Otherwise, all the participants had normal smell of vaginal secretion, normal vaginal appearance, no menstrual bleeding, and no reddening of the vulva and/or vagina.

There were 34 AEs associated with the intervention recorded in 21 participants. These were balanced equally between the verum (17 events in 11 participants) and placebo (17 events in 10 participants) groups. A total of 11 AEs were classified as potentially related to the IP (for three participants in the placebo group and four participants in the verum group). The most common organ class affected by AEs was the gastrointestinal system (total 14 events). These were equally divided between verum (7) and placebo (7). There were no severe AEs or serious adverse events and no events requiring withdrawal from the study.

## 4. Discussion

A BV-associated microbiota resembles that of CST IV, with pronounced abundance of members from the genera *Gardnerella, Atopobium*, and *Prevotella*, whereas lactobacilli-predominance is associated with a healthy-like vaginal microbiota [7,9,10]. Both species richness and diversity elevate with BV and recurrent BV [7,10]. The Nugent score (0–10), which is used for BV diagnosis, correlates negatively with lactobacilli abundance and positively with microbial diversity [7]; when BV is treated with an antibiotic (5-day metronidazole course), lactobacilli numbers start to elevate, especially *L. iners*, and the microbial diversity (α-diversity) is restored even within 8 days, with no significant differences between relative abundances of bacterial taxa remaining after 15 days.

These depleted vaginal lactobacilli levels, associated with BV and increased disease risk [58,59], can potentially be normalised with orally consumed probiotics [23,24,25,26], though further systematic research on the efficacy and safety of strain specific effects of probiotics is warranted [60]. For the probiotic strains applied for vaginal health, the ability to locally colonise the vagina has been considered a beneficial trait, although the colonisation may be transient and the mode of action in vivo remains obscured [20,21]. Thus, our objective in the present study was to assess the vaginal colonisation potential of two orally consumed probiotic strains in premenopausal women without vaginal complaints, and to evaluate whether there were any intervention-related alterations on a well-balanced commensal vaginal microbiota or immune marker profile.

Vaginal colonisation of La-14 or HN001 on species or strain level was not detected with the applied methods allowing detection of 5.29 and 5.11 Log10 genomes per swab, respectively (Table 5). Previously La-14 and HN001 have been shown to elevate vaginal *L. acidophilus* and *L. rhamnosus* levels in a comparable study setting with calculations based either on all DNA extracted from the vaginal swab (per swab) or on mass of DNA (per 100 ng of DNA) [23,30], neither allowing inference of the actual target DNA quantities per swab. In the study by Russo and colleagues [23], the baseline and end-of-intervention quantities can be estimated to have been approximately 2.2 and 2.8 Log10 genomes per ng of DNA for *L. rhamnosus* and 3.3 and 3.9 Log10 genomes per ng of DNA for *L. acidophilus*, respectively [23]. For orally supplemented *Lacticaseibacillus paracasei* LPC-S01, the vaginal colonisation on strain level was detected at approximately 4–6 logs below the level of total bacteria present in a vaginal sample, ranging up to approximately 5 Log10 cells/swab [27]. Moreover, Hertz and colleagues have published a non-controlled clinical study assessing the vaginal microbiota from 16 women with self-reported good health during oral probiotic consumption (*L. rhamnosus* PB01 and *L. gasseri* EB01) applying shotgun metagenomics [61]. From the intervention period samples, with discriminatory single nucleotide polymorphisms (DSNPs), supplemented strain associated reads were detected from 3/16 participants at or below 1.4% of the supplemented species-associated reads or 0.004% of the total microbiota, suggesting that if the strains were present, their abundance was very low. Unfortunately, the baseline samples were not assessed for DSNPs and there was no placebo control. However, comparison between units, studies, and even samples within a study is hindered by the high variance in DNA extraction efficiency from the vaginal swabs seen in our study (up to 150-fold difference) and in previous studies [56]. Regardless, we would also have expected to be able to detect potential vaginal colonisation with the assays we optimised and applied in our study, although at a low level.

For the *L. rhamnosus* assay we applied the same primers as were used in prior studies [23,30,38], but with a higher annealing temperature. For *L. acidophilus,* we applied a different assay [44] than the one used in prior studies [23,30,38] due to a persistent double-peak dissociation curve generated from a *L. acidophilus* standard (DGCC 8698 and La-14 both tested as the standard strain through extensive reaction condition optimisation steps) suggesting suboptimal performance of the earlier applied assay, i.e., that there were either two different amplicons being generated during the qPCR, or that the amplicon had a secondary DNA structure leading to a step-wise dissociation. The assay did, however, amplify DNA extracted from the clinical study vaginal swab samples and from pure cultures of *L. crispatus, L. gasseri,* and *L. jensenii,* resulting in a uniform single-peak dissociation curve. Due to a difference in the melting point temperatures of these two PCR products, quantification of the samples with *L. acidophilus* as the standard was not possible with SYBR chemistry. However, when we changed the standard to *L. crispatus* (likely *L. jensenii* or *L. gasseri* would have performed similarly as a standard), we were able to quantify the clinical samples (Table 6), Our results resemble those published by De Alberti and colleagues [30], albeit without showing a significant difference between groups, potentially due to the very high vaginal lactobacilli levels confirmed already present at baseline in our study. Moreover, De Alberti and colleagues detected *L. rhamnosus* and *L. acidophilus* in both groups: placebo and verum [30]. The prior clinical studies detecting vaginal colonisation of *L. acidophilus* and *L. rhamnosus* after La-14 and HN001 consumption [23,30] do mention confirming the dissociation curve quality visually, but not comparing it with a standard. Thus, it remains unclear whether the detected elevation from baseline in the study by De Alberti et al. [30] and Russo et al. [23] could have reflected an increase in commensal *Lactobacillus* spp., including *L. crispatus*, *L. gasseri*, *L. jensenii,* and alike, rather than an increase solely of the supplemented species, *L. acidophilus*. The former would potentially have an even more significant health benefit, and, indeed, the product tested has been shown to reduce the Nugent score indicating an increase in commensal *Lactobacillus* spp. [23]. Moreover, the 50 mg of lactoferrin included in the Respecta^®^ complex [23,30] may also contribute to the difference between groups in vaginal lactobacilli levels: in a 30-day pilot intervention study on pregnant women, a four times higher daily dose of orally supplemented lactoferrin without probiotics was shown to increase the prevalence of a normal vaginal microbiota, defined as the predominance of variable size lactobacilli in phase contrast microscopy of a vaginal swab [37].

We had recruited participants devoid of vaginal complaints (Nugent score 0–3 and vaginal pH ≤ 4.5) into an intervention with lactobacilli being orally supplemented as the test ingredient expecting the vaginal pH and microbiota to remain relatively stable. Both outcomes were followed to assess the safety of the verum IP and potential intervention-related fluctuations in a set-up not assessing treatment or prevention. The vaginal pH remained stable and at a healthy level throughout the study, being ≤ 4.5 for almost all participants in both groups at all study visits, although no statistically significant changes within a healthy pH range were detected (Supplementary Table S1; Table 7). The pH results corresponded with De Alberti et al. [30]. The vaginal microbiota was lactobacilli predominant in both groups, with no intervention related changes detected (Figure 3; Supplementary Table S2); this was also reflected by the high prevalence of lactobacilli dominated CSTs (I, II and V; Table 8). Numerically intriguing results were observed between groups for the percentage of participants with >1% reduction in *G. vaginalis* and *L. jensenii* ASVs for the benefit of the verum IP, but the difference was not statistically significant (Table 9). The positive correlation noted between non-lactobacilli species but not among different *Lactobacillus* spp. can be seen as characteristic of vaginal microbiota, which tend to be either a mixed type (CST IV) or predominated by one *Lactobacillus* species (CST types I, II, III, and IV) [1]. BMI, which had a wide range at baseline in the current study (Table 3), was found to correlate positively with *G. vaginalis* and *C. ureolyticus*, in alignment with earlier findings by Allen and colleagues [62]. Thus, restricting BMI range at recruitment or stratifying for BMI at randomisation could be advisable in future studies assessing vaginal microbiota.

The noted wide range in DNA extraction efficiency was likely due to varying amounts of host DNA carryover during DNA extraction and may have challenged detection of intervention-emergent changes among less abundant ASVs. DNA extracted from vaginal swab samples may contain over 95% of host DNA if host DNA is not removed during the extraction process [63]. As an example, 90% host DNA carryover already prevents detection of 16S rDNA reads that are present as 0.1–1% of total reads in a metagenomic sequence analysis [64]. Indeed, DNA extraction efficiency has been inversely linked with 16S rDNA sequencing-based microbial diversity in a study comparing different DNA extraction methods for vaginal swabs [65]. In our study, colonisation was not detected with the sequencing analysis either. For *L. acidophilus*, this would not have been possible as the 16S rDNA V4 region does not differentiate between *L. acidophilus* and *L. crispatus*, but since the V4 region distinguishes species relevant for vaginal microbial disturbances (e.g., *G. vaginalis*), it was selected.

We had aimed to repeat the results published by De Alberti and colleagues, reporting elevation of *L. acidophilus* and *L. rhamnosus* among women with no vaginal complaints during a 2-week intervention with La-14 and HN001 [30]; thus, we also recruited participants devoid of vaginal symptoms. To add a non-subjective measure for vaginal health, we used Nugent score (0–3) as an inclusion criterion, although it had not been applied in the prior study. In our study, Nugent score was not measured over the course of the study, but both vaginal pH and the sequencing results indicate healthy vaginal microbiota throughout the intervention. A prior observational study assessing the vaginal microbiota before and after a 5-day metronidazole treatment for BV and that of healthy controls has shown a clear increase in the vaginal microbiota diversity already at Nugent 1–3 in comparison to Nugent 0 [7]. By chance, the screening visit Nugent scores of the eligible randomised participants were predominantly 0 (Nugent was 0 for 79.2% and 64.0% of verum and placebo participants, respectively), although Nugent 0–3 was permissible. Consequently, the study population in our study may have been especially challenging for detecting colonisation. Even when locally applied at a daily dose of 2 × 10^6^ or 2 × 10^8^ CFU for three days, *L. crispatus* CTV-05 colonisation has been shown to be significantly more likely during weekly follow-up visits among women initially devoid of commensal *L. crispatus* within their vaginal microbiota and not practicing unprotected vaginal intercourse [66]. Additionally, with vaginally applied probiotics, Marcotte and colleagues have reported colonisation to be more efficient among women with BV receiving antibiotic treatment than among women within the healthy control group devoid of BV and not subject to an antibiotic course [67], suggesting that perturbations in the vaginal microbiota enhance the colonisation efficiency of vaginally applied probiotics. Oral supplementation predisposes the vagina to much lower quantities of the supplemented strain(s) than local application; thus, comparable competitive and environmental factors may play an even more significant role. Although *L. acidophilus,* being a gut-oriented lactobacillus, adapts well to simulated vaginal fluid, it would unlikely be able to successfully compete with abundant endogenous vaginal lactobacilli such as *L. crispatus, L. gasseri,* and *L. jensenii* [68]. Aligned, Chen and colleagues found the effects of an oral probiotic to be more probable in the vaginal microbiota of participants with higher intraindividual variability within the vaginal microbiome already before the intervention commenced, whereas participants with a stable and lactobacilli-rich vaginal microbiota at baseline were more resilient to the effects of the probiotic [33]. The study followed 60 Chinese female participants for over a year with multiple baseline samples, and intervention and follow-up samples collected from the oral cavity, feces, and vagina. Colonisation was not shown; rather, a potential for the increase of commensal vaginal lactobacilli was indicated [33]. A further unplanned confounder was that our study took place between November 2020 and March 2021 in Glasgow, UK, while everyday life was greatly restricted due to the Covid-19 pandemic and participants were mostly home bound and obligated to social distancing. This almost certainly resulted in reducing intrusions to microbial balance, also in the vagina, due to less social and environmental contact, elevated hygiene practices, and potentially a less versatile diet [69], and may have further enhanced the resilience of participants to probiotic colonisation above detection limit.

The current study also provides insights on the effects of probiotics on the mucosal immune system modulation in a healthy vaginal tract. The participants in the verum group showed statistically significant decrease in elafin and HBD-3, with a similar trend in SLPI, when compared with the placebo (Table 10). Interestingly, reduction in vaginal SLPI has also been observed, albeit in vitro, with another strain: *L. rhamnosus* Lcr35 [70]. According to the literature, it is well established that the presence of *Lactobacillus* spp. in healthy vaginal microbiota has an important role in competitive exclusion of pathogenic bacteria, competition for nutrients, production of antimicrobial substances, and on the immune system [59,71]. In this context, Jiang et al. [72] showed a significant correlation between HBD-2 and HBD-3 and DNA levels of *L. jensenii*, as well as HBD-2 and DNA levels of *L. crispatus*, in cervicovaginal lavage samples from healthy women. In our study, we found significant correlation of HBD-3 with *L. jensenii* and of IgA with *L. crispatus* in 40% of participants irrespective of intervention/group (Table 11). In another study, Orfanelli et al. [73] investigated the association of SLPI concentrations with CST in healthy women. They showed that median levels of SLPI was three times higher when the vaginal microbiota was dominant with *L. crispatus* than when it was dominant with *L. iners*. While analyzing the concentration of immune markers at baseline, combined for the verum and the placebo, we also observed a trend of higher SLPI and lower HBD-3 in CST I compared to CST III (Supplementary Figure S3). The other CSTs were not accounted for due to their low prevalence in the population. As mentioned above, commensal bacteria in the vagina represent one of the first lines of defense against vaginal infection, and some *Lactobacillus* species, in particular *L. crispatus*, play important roles in this defense, thus regulating the vaginal immune system [6]. Hence, the small decrease in immune marker levels of HBD-3 and SLPI in the verum group (Table 10) could hypothetically reflect a small change of *Lactobacillus* species composition (or metabolites produced) within the population that we could not detect with 16S sequencing (Figure 3). Indeed, in healthy vaginal mucosa, probiotics more likely promote immunological homeostasis rather than inflammation. Given the small sample size, large standard deviation, and stable vaginal health and microbiota, further exploration in a more plausible setting is needed to have a better understanding of the intervention effect on the immune markers.

Taken together, the high and constant lactobacilli predominance observed in the vaginal swab samples of the current study likely left less of a niche for non-commensal lactobacilli strains to colonise the vagina and increased the technical challenges in detecting any potential colonisation, if such existed. DNA extracted from vaginal swab samples are bound to have high but varying amounts of host DNA carryover and, if from healthy volunteers, commensal microbiota likely consisting predominantly of lactobacilli. This underlies the need for stringent optimisation procedures and interpretation of results when PCR-based analyses are applied to detect low levels of supplemented lactobacilli. The stability of the healthy-like vaginal microbiota and pH throughout the study highlights the safety of using La-14 and HN001 as over-the-counter supplements without the risk of jeopardising well-balanced vaginal microbiota. Vaginal immune markers, elafin, HBD-3, and SLPI, should be evaluated further in future probiotic intervention studies elucidating the effects of the supplementation on vaginal health. No safety concerns were raised regarding vital signs, menstruation, vaginal health, or AEs.

## 5. Conclusions

Colonisation of La-14 or HN001 was not observed above the detection limit 5.29 and 5.11 Log10 genomes per vaginal swab, respectively. Oral supplementation of La-14 and HN001 at 10^10^ CFU in a 4:1 ratio for two weeks had no aberrant effects on stable and healthy commensal vaginal microbiota and immunological homeostasis.

## Figures and Tables

**Table 1:** Inclusion and exclusion criteria.

Inclusion Criteria	Exclusion Criteria
Female Age 18–50 years (fertile age) Caucasian No vaginal infections within previous 6 months Has not participated in another investigational drug clinical trial within 1 month (30 days) or having received an investigational drug within the last month (30 days) before the start of screening No significant changes in daily routines related to dietary/activity patterns Willingness to take dietary supplements Valid contraception for the duration of the study Willingness to collaborate in completing the binding parts of the study protocol	Vaginal or urinary complaints Vaginal pH > 4.5 Pregnant or planning pregnancy Breast feeding History of vulvovaginal pathological conditions Antibiotic usage during last 3 months Oral corticosteroid usage during last 3 months Use of vulvovaginal medication Acquired or congenital immune deficiency Recent history of radiotherapy Prolonged use of corticosteroids or other immune modulating medication Habitual use of probiotic supplementation Menstrual irregularities including menopause On-going diagnosed disease which, in the opinion of the investigator, makes the participant unfit for the study Intolerance to any of the study products History of alcohol abuse within 2 years History of drug abuse within 2 years Unable to communicate with the investigator Nugent score > 3 (sampled during screening visit)

**Table 2:** Quantitative PCR (qPCR) assays applied to assess colonisation of supplemented strains from vaginal swab samples. F, forward primer; R, reverse primer; P, probe.

Primer/Probe (5′–3′)	Master Mix	Annealing Temperature
Primary outcome assays		
*L. acidophilus* (species level) [44]	1 × SYBR FAST	60 °C
F: CCTTTCTAAGGAAGCGAAGGAT (400 nM)		
R: ACGCTTGGTATTCCAAATCG (400 nM)		
*L. rhamnosus* (species level) [45]	1 × SYBR FAST	62 °C
F: TGCTTGCATCTTGATTTAATTTTG (400 nM)		
R: GGTTCTTGGATYTATGCGGTATTAG (400 nM)		
Exploratory outcome assays		
*L. acidophilus* La-14 (strain level) [46]	1 × Taq FAST Advanced	60 °C
F: CCGGTTAATAAAATCTTTTCACCTTG (600 nM)		
R: GCAGTTATTAATCGTGATTTGCATATAAATT (600 nM)		
P: FAM-AGTTGATCAGTCAGCAAGTAGTGTTATGG-IowaBlack (300 nM)		
*L. rhamnosus* HN001 (strain level)	1 × Taq FAST Advanced	60 °C
F: CTGGAGGAGATCACAACGACT (400 nM)		
R: ATTGTCCCAACGCTGAATGC (400 nM)		
P: FAM-TGAAGACAAGGTTGCGCCCTGTACACTGTTA-IowaBlack (200 nM)		
Post hoc analysis		
*L. acidophilus* (species level) [47]	1 × SYBR FAST	62 °C
F: TGCAAAGTGGTAGCGTAAGC (400 nM)		
R: CCTTTCCCTCACGGTACTG (400 nM)		

**Table 3:** Demographic characteristics for the intention-to-treat population. BMI, body mass index.

Item	Statistic	Placebo (n = 24)	Verum (n = 25)
Age (years)	Mean (SD)	30.9 (6.89)	32.4 (7.94)
	Median	30.0	33.0
	Min, Max	21, 41	20,47
BMI (kg/m^2^)	Mean (SD)	26.9 (6.94)	26.5 (6.41)
	Median	25.3	23.8
	Min, Max	16.8, 41.5	19.9, 43.6
Smoking status			
Every day smoker	n (%)	0 (0.0)	2 (8.0)
Former smoker	n (%)	7 (29.2)	3 (12.0)
Never smoker	n (%)	16 (66.7)	16 (64.0)
Someday smoker	n (%)	1 (4.2)	4 (16.0)
Alcohol use			
No	n (%)	4 (16.7)	4 (16.0)
Yes	n (%)	20 (83.3)	21 (84.0)
Contraceptive method			
Hormonal	n (%)	14 (58.3)	9 (36.0)
Not hormonal	n (%)	10 (41.7)	16 (64.0)
Nugent score			
0	n (%)	19 (79.2)	16 (64.0)
1	n (%)	3 (12.5)	3 (12.0)
2	n (%)	2 (8.3)	2 (8.0)
3	n (%)	0 (0.0)	3 (12.0)

**Table 4:** Details on menstruation cycle and obstetric history for the intention-to-treat population.

Item	Statistic	Placebo (n = 24)	Verum (n = 25)
Duration of menstruation (days)	n	18	19
	Mean (SD)	4.83 (1.15)	4.53 (1.07)
	Median	5.00	5.00
	Min, Max	3.00, 7.00	2.00, 7.00
			
Length of menstrual cycle (days)	n	18	19
	Mean (SD)	27.61	28.47
	Median	28.00	28.00
	Min, Max	24.00, 29.00	25.00, 35.00
			
Menarche age (years)	n	24	25
	Mean (SD)	12.63 (1.53)	13.04 (1.59)
	Median	12.50	13.00
	Min, Max	10.00, 16.00	10.00, 16.00
			
		n (%)	n (%)
Intermenstrual bleeding	N/A	5 (20.8)	5 (20.0)
	No	19 (79.2)	20 (80.0)
			
Menstrual flow	Mild	6 (25.0)	5 (20.0)
	Moderate	12 (50.0)	14 (56.0)
			
Post-coital bleeding	No	24 (100.0)	25 (100.0)
			
Leucorrhea	No	23 (95.8)	25 (100.0)
	Yes	1 (4.2)	0 (0.0)
			
Number of pregnancies	0	14 (58.3)	12 (48.0)
	1	4 (16.7)	3 (12.0)
	2	5 (20.8)	7 (28.0)
	3	1 (4.2)	3 (12.0)
			
Operations	No	22 (91.7)	23 (92.0)
	Yes	2 (8.3)	2 (8.0)
			
Pain in the lower abdomen	No	24 (100.0)	25 (100.0)
			
Pelvic or abdominal pelvic pain	No	24 (100.0)	24 (96.0)
	Yes	0 (0.0)	1 (4.0)
			
Presence of vaginal inflammations, dryness, itchiness, stinging	No	24 (100.0)	25 (100.0)
			
Sterility	No	24 (100.0)	25 (100.0)
			
Vulvovaginal infections	No	24 (100.0)	25 (100.0)

**Table 5:** Vaginal colonisation of *Lactobacillus acidophilus* and *Lacticaseibacillus rhamnosus* at species level shown as mean (SD) log 10 genomes/swab. The limit of detection (LOD) values were 5.29 log 10 genomes/swab and 5.11 log 10 genomes/swab for *L. acidophilus* and *L. rhamnosus*, respectively. V, Visit; V2, baseline visit; V3, after one week of intervention; V4, after two weeks of intervention; V5, after a one-week follow-up.

Assay	Visit	Placebo (n = 20–23)	Verum (n = 23–25)
Genomes detected		log 10 genomes/swab	log 10 genomes/swab
*L. acidophilus*	V2	<5.29 (0.00)	<5.29 (0.00)
	V3	<5.29 (0.00)	<5.29 (0.00)
	V4	<5.29 (0.00)	<5.29 (0.00)
	V5	<5.29 (0.00)	<5.29 (0.00)
*L. rhamnosus*	V2	5.12 (0.056)	<5.11 (0.000)
	V3	<5.11 (0.017)	<5.11 (0.000)
	V4	<5.11 (0.009)	<5.11 (0.000)
	V5	5.12 (0.048)	<5.11 (0.000)

**Table 6:** Percentage of participants with an over 2-fold increase from baseline in lactobacilli copies per ng of DNA or swab. The analysis applied *Lactobacillus acidophilus* primers designed by Song et al., 2000, with *Lactobacillus crispatus* DSM 20,584 used as the standard. V, Visit; V3, after one week of intervention; V4, after two weeks of intervention.

Group	% (*n*/*n*) of Participants with > 2-Fold Increase from Baseline in Genome Copies/ng		% (*n*/*n*) of Participants with > 2-Fold Increase from Baseline in Genome Copies/swab	
	V3	V4	V3	V4
Verum	36 (9/25)	40 (10/25)	40 (10/25)	36 (9/25)
Placebo	30 (7/23)	15 (3/20)	26 (6/23)	30 (6/20)

**Table 7:** Prevalence of change in vaginal pH during the intervention and follow-up measured with NutraBlast^®^ Feminine pH Test Strip (NutraBlast, Pompano Beach, FL, USA) on each visit. V, Visit; V3, after one week of intervention; V4, after two weeks of intervention; V5, after a one-week follow-up.

Visit	Categorised Change in Vaginal pH from Baseline	Placebo (n = 22–24)	Verum (n = 23–25)
		n (%)	n (%)
V3	Decrease	5 (20.8)	5 (20.0)
	No change	13 (54.2)	16 (64.0)
	Increase	6 (25.0)	4 (16.0)
V4	Decrease	4 (16.7)	8 (32.0)
	No change	14 (58.3)	14 (56.0)
	Increase	4 (16.7)	3 (12.0)
V5	Decrease	2 (8.3)	10 (40.0)
	No change	16 (66.7)	8 (32.0)
	Increase	5 (20.8)	5 (20.0)

**Table 8:** Community state type (CST) distribution of sequenced samples per group and visit. V, visit; V2, baseline visit; V3, after one week of intervention; V4, after two weeks of intervention; V5, after a one-week follow-up.

Group	Visit	Samples	CST I	CST II	CST III	CST IV-B	CST IV-A	CST V
		n	n (%)	n (%)	n (%)	n (%)	n (%)	n (%)
Verum	All visits	90	38 (42.2)	3 (3.3)	26 (28.9)	3 (3.3)	5 (5.6)	15 (16.7)
	V2	23	9 (39.1)	1 (4.3)	8 (34.8)	1 (4.3)	1 (4.3)	3 (13.0)
	V3	23	8 (34.8)	1 (4.3)	5 (21.7)	1 (4.3)	3 (13.0)	5 (21.7)
	V4	22	10 (45.5)	1 (4.5)	7 (31.8)	0 (0.0)	0 (0.0)	4 (18.2)
	V5	22	11 (50.0)	0 (0.0)	6 (27.3)	1 (4.5)	1 (4.5)	3 (13.6)
								
Placebo	All visits	86	56 (65.1)	4 (4.7)	14 (16.3)	1 (1.2)	2 (2.3)	9 (10.5)
	V2	22	13 (59.1)	1 (4.5)	4 (18.2)	1 (4.5)	0 (0.0)	3 (13.6)
	V3	21	14 (66.7)	1 (4.8)	3 (14.3)	0 (0.0)	0 (0.0)	3 (14.3)
	V4	20	14 (70.0)	1 (5.0)	3 (15.0)	0 (0.0)	1 (5.0)	1 (5.0)
	V5	23	15 (65.2)	1 (4.3)	4 (17.4)	0 (0.0)	1 (4.3)	2 (8.7)

**Table 9:** Percentage of participants with over 1% change in relative abundance of amplicon sequence variants (ASVs) assigned to predominant *Lactobacillus* species or *Gardnerella vaginalis*. V, Visit; V3, after one week of intervention; V4, after two weeks of intervention; V5, after a one-week follow-up.

Group	Visit	*N*	*G* *ardnerella vaginalis*	*Lactobacillus* spp.	*Lactobacillus crispatus*	*Lactobacillus jensenii*	*Lactobacillus gasseri*	*Lactobacillus iners*
Percentage of participants with >1% increase in the relative abundance of an ASV. n (%)								
Verum	V3	23	4 (17)	3 (13)	5 (22)	7 (30)	3 (13)	4 (17)
	V4	22	3 (14)	4 (18)	7 (32)	7 (32)	2 (9)	6 (27)
	V5	22	4 (18)	2 (9)	8 (36)	5 (23)	0 (0)	7 (32)
Placebo	V3	20	4 (20)	3 (15)	5 (25)	4 (20)	3 (15)	3 (15)
	V4	19	2 (11)	4 (21)	8 (42)	2 (11)	2 (11)	4 (21)
	V5	22	4 (18)	3 (14)	7 (32)	5 (23)	2 (9)	2 (9)
Percentage of participants with >1% decrease in the relative abundance of an ASV. n (%)								
Verum	V3	23	4 (17)	2 (9)	6 (26)	1 (4)	1 (4)	9 (39)
	V4	22	5 (23)	1 (5)	5 (23)	2 (9)	0 (0)	7 (32)
	V5	22	5 (23)	1 (5)	3 (14)	4 (18)	3 (14)	7 (32)
Placebo	V3	20	0 (0)	2 (10)	6 (30)	4 (20)	0 (0)	4 (20)
	V4	19	1 (5)	1 (5)	5 (26)	5 (26)	1 (5)	3 (16)
	V5	22	2 (9)	3 (14)	7 (32)	6 (27)	1 (5)	5 (23)

**Table 10:** The effect of the supplementation on vaginal immune markers during the intervention (V3 and V4) and follow-up (V5). HBD, Human β-defensin; IgA, Immunoglobulin A; SLPI, Secretory leukocyte protease inhibitor; V, Visit; V3, after one week of intervention; V4, after two weeks of intervention; V5, after a one-week follow-up.

Analysis Method	Parameter	Visit	Verum vs. Placebo	*p*
RM-ANCOVA (log-transformed)	SLPI (*p* = 0.15)	V3	−0.194 (−0.793, 0.406)	0.52
		V4	−0.148 (−0.657, 0.362)	0.56
		V5	−0.488 (−0.988, 0.013)	0.056
	Elafin (*p* = 0.022)	V3	−0.310 (−0.648, 0.028)	0.071
		V4	−0.305 (−0.626, 0.015)	0.062
		V5	−0.318 (−0.640, 0.004)	0.051
	IgA (*p* = 0.82)	V3	0.038 (−0.401, 0.477)	0.86
		V4	−0.079 (−0.577, 0.420)	0.75
		V5	−0.083 (−0.625, 0.460)	0.76
RM-ANCOVA (square-root-transformed)	HBD-1 (*p* = 0.97)	V3	−1.48 (−11.78, 8.83)	0.77
		V4	2.50 (−7.42, 12.43)	0.61
		V5	−1.51 (−9.40, 6.38)	0.70
	HBD-3 (*p* = 0.028)	V3	−6.34 (−24.72, 12.05)	0.49
		V4	−13.86 (−31.69, 3.97)	0.12
		V5	−22.70 (−39.03, −6.37)	0.008

**Table 11:** Repeated measures correlations between normalised reads of 16 ASVs that were present in at least 40% of the participants and immune markers. The verum and placebo groups and all timepoints were joined for the analysis. Only significant results are shown. HBD, Human β-defensin; IgA, Immunoglobulin A; NS, not significant.

	*Finegoldia magna*	*Gardnerella vaginalis*	*Lactobacillus crispatus/acidophilus*	*Lactobacillus* *jensenii*	*Prevotella* *bivia*
	Average R-Value (*p*)				
HBD-1	−0.173 (0.05)	NS	NS	NS	−0.231 (0.01)
HBD-3	−0.2 (0.02)	−0.197 (0.03)	NS	−0.193 (0.03)	NS
IgA	NS	NS	−0.258 (0.004)	NS	NS

## Data Availability

Data generated during the study is not publicly available due to lack of consent from the study participants. The study is registered at https://www.isrctn.com/ (accessed on 14 February 2023) under identifier ISRCTN29375062.

## Supplementary Materials

The following supporting information can be downloaded at: https://www.mdpi.com/article/10.3390/microorganisms11020499/s1, Table S1: Prevalence of vaginal pH results measured with NutraBlast^®^ Feminine pH Test Strip (NutraBlast, Pompano Beach, FL, USA ) on each visit; Table S2: Average, SD, minimum and maximum relative abundance values of amplicon sequence variants (ASVs) detected in at least 2 participants per group and visit in the 16S rDNA sequencing analysis; Figure S1: Immune marker box plots by group and visit; Figure S2: Significant correlations detected between immune markers and amplicon sequence variants (ASVs); Figure S3: Immune marker concentrations at baseline for community state types (CSTs).
