# Supplementary material for: A Healthy Vaginal Microbiota Remains Stable during Oral Probiotic Supplementation: A Randomised Controlled Trial
Source: Microorganisms. 2023 Feb 16;11(2):499. doi: 10.3390/microorganisms11020499 (PMC9961720; doi:10.3390/microorganisms11020499)
Supplement: Supplementary file 1 [file microorganisms-11-00499-s001.zip › Lyra et al Supplementary Tables and Figures_20230123.pdf]

Supplementary Tables and Figures

Table S1. Prevalence of vaginal pH results measured with NutraBlast® Feminine pH Test Strip on each visit.

Group	pH*	Visit 2	Visit 3	Visit 4	Visit 5
Verum	n	25	25	25	23
	3.0	0 (0.0)	0 (0.0)	0 (0.0)	0 (0.0)
	3.5	1 (4.0)	0 (0.0)	1 (4.0)	0 (0.0)
	4.0	11 (44.0)	14 (56.0)	17 (68.0)	16 (69.6)
n (%)	4.5	12 (48.0)	11 (44.0)	7 (28.0)	5 (21.7)
	5.0	1 (4.0)	0 (0.0)	0 (0.0)	2 (8.7)
	5.5	0 (0.0)	0 (0.0)	0 (0.0)	0 (0.0)
Placebo	n	24	24	22	23
	3.0	0 (0.0)	0 (0.0)	0 (0.0)	0 (0.0)
	3.5	1 (4.2)	1 (4.2)	1 (4.5)	0 (0.0)
	4.0	18 (75.0)	16 (66.7)	16 (72.7)	15 (65.2)
n (%)	4.5	4 (16.7)	7 (29.2)	5 (22.7)	8 (34.8)
	5.0	1 (4.2)	0 (0.0)	0 (0.0)	0 (0.0)
	5.5	0 (0.0)	0 (0.0)	0 (0.0)	0 (0.0)

*The NutraBlast® Feminine pH Test Strip is interpreted with a scale for pH 3.0, 3.5, 4.0, 4.5, 5.0 and 5.5.

Table S2 (Average, SD, minimum and maximum relative abundance values of amplicon sequence variants (ASVs) detected in at least 2 participants per group and visit in the 16S rDNA sequencing analysis.) is supplied as a separate Excel file.

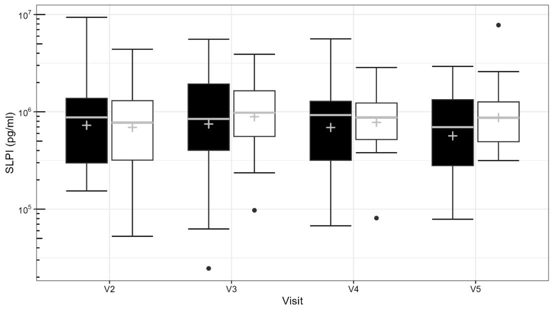

a) SLPI

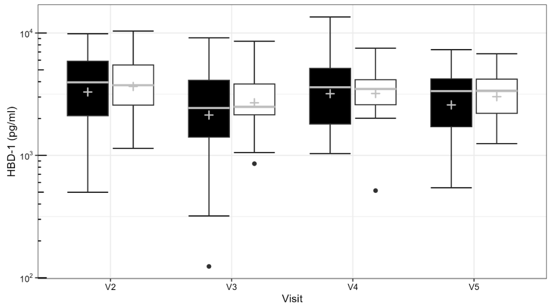

d) HBD-1

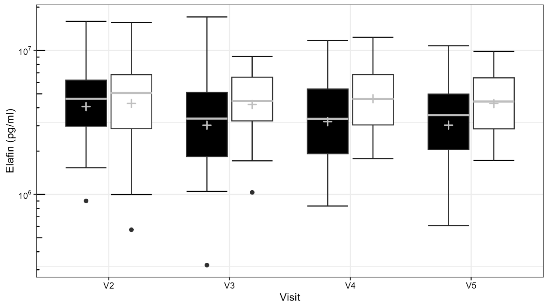

b) Elafin

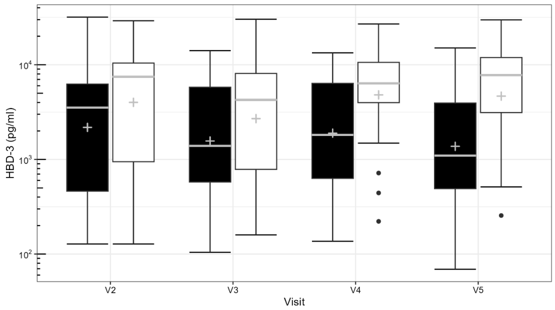

e) HBD-3

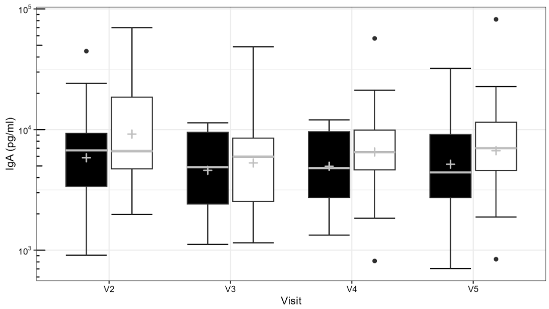

c) IgA

Figure S1. Immune marker box plots by group and visit. The verum is presented with black and the placebo with white colour. HBD; Human β-defensin. IgA; Immunoglobulin A. SLPI; Secretory leukocyte protease inhibitor.

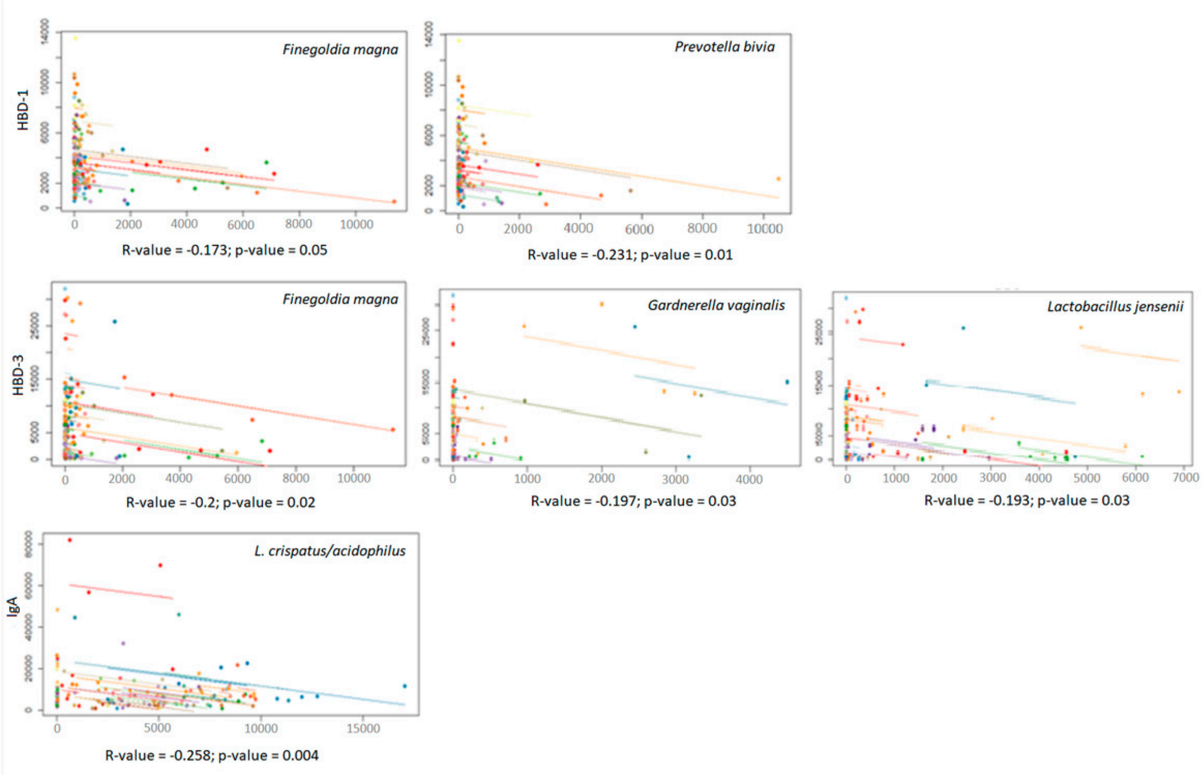

Figure S2. Significant correlations detected between immune markers and amplicon sequence variants (ASVs). HBD; Human β-defensin. IgA; Immunoglobulin A. *L. crispatus/acidophilus; Lactobacillus crispatus/acidophilus.*

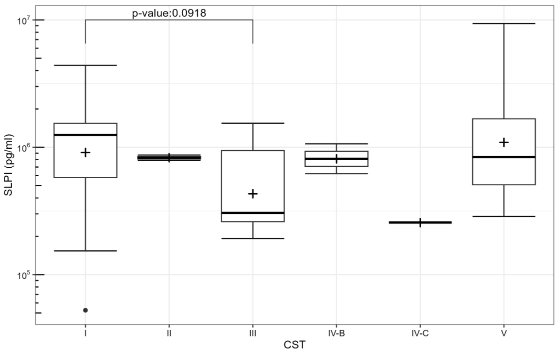

a) SLPI

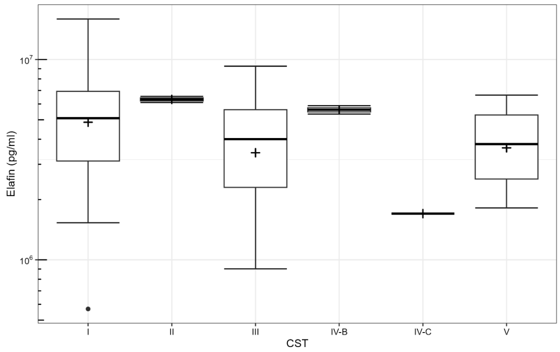

b) Elafin

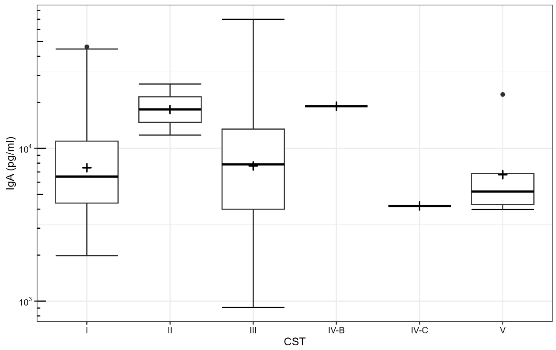

c) IgA

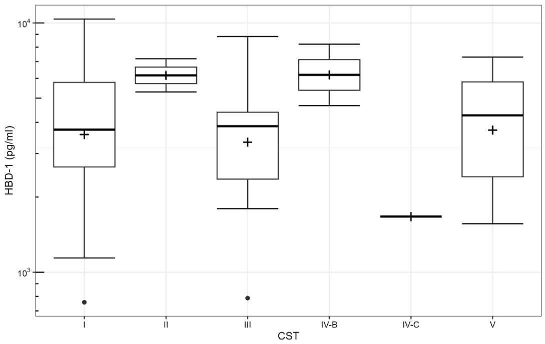

d) HBD-1

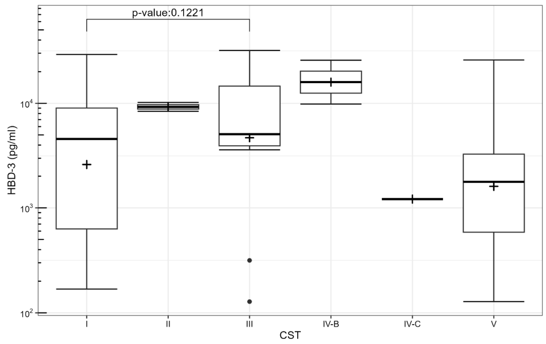

e) HBD-3

Figure S3. Immune marker concentrations at baseline for community state types (CSTs). For Secretory leukocyte protease inhibitor (SLPI) and Human β-defensin 3 (HBD-3) there was a trend of difference between CST I (dominated by *Lactobacillus crispatus*) and CST III (dominated by *Lactobacillus iners*). IgA; Immunoglobulin A.
